# Retinal connections to migraine

**DOI:** 10.3389/fneur.2026.1835914

**Published:** 2026-07-02

**Authors:** Michal Fila, Jan Krekora, Jarosław Drożdż, Kai Kaarniranta, Janusz Blasiak

**Affiliations:** 1 Department of Developmental Neurology and Epileptology, Polish Mother’s Memorial Hospital Research Institute, Lodz, Poland; 2 2nd Department of Cardiology, Medical University of Lodz, Lodz, Poland; 3 Department of Ophthalmology, University of Eastern Finland, Kuopio, Finland; 4 Department of Ophthalmology, Kuopio University Hospital, Kuopio, Finland; 5 Faculty of Medicine, Collegium Medicum, Mazovian Academy in Plock, Plock, Poland

**Keywords:** age-related macular degeneration, AMD, migraine, neural retina, photophobia, retinal migraine, retinal nerve fiber layer thickness, retinal pigment epithelium

## Abstract

**Objective:**

This narrative review aims to examine potential associations between migraine and retinal disorders, with particular attention to retinal migraine, age-related macular degeneration (AMD), retinal vascular occlusions, and photophobia.

**Background:**

The role of retinal pathways in migraine pathophysiology remains poorly understood. As a component of the central nervous system, the retina provides a unique and accessible window into neuronal structure and function. Emerging observations suggest that structural and functional retinal changes may occur in individuals with migraine; however, their relevance and consistency across studies remain uncertain.

**Results:**

Visual phenomena are characteristic of migraine aura, yet the extent to which migraine affects retinal structure and function is still unclear. Retinal migraine, while suggestive of a direct retinal involvement, is rare and insufficiently characterized to support mechanistic conclusions. Epidemiological data indicate that individuals with migraine may have an increased risk of neovascular AMD, raising the possibility of shared pathogenic pathways. In addition, photophobia, retinal artery occlusion, and alterations in retinal nerve fiber layer thickness have been reported more frequently in migraine patients than in controls, although causality has not been established. Mechanisms implicated in AMD, including microvascular dysfunction, impaired DNA damage response, disrupted autophagy, and mitochondrial dysregulation, have also been proposed in migraine, but evidence remains limited.

**Conclusion:**

Migraine has been linked to several retinal conditions, with AMD showing the most consistent association. While overlapping biological processes are suggested, further studies are needed to clarify their significance and determine whether retinal alterations contribute to migraine pathophysiology or represent secondary phenomena.

## 1 Introduction

The human retina captures light, converts it into an electrical signals, and transmits these signals to the brain for visual processing. Retinal homeostasis is maintained through complex interactions among the central nervous system (CNS), circulation, endocrine, and immune systems (1). Disruptions in retinal homeostasis have been associated with a range ofretinal disorders, including age-related macular degeneration (AMD), retinitis pigmentosa, diabetic retinopathy, and other complex diseases that could cause irreversible visual impairments, including permanent vision loss (2).

One of the conditions classified within the International Classification of Headache Disorders (ICHD-3), retinal migraine (code 1.2.4) (3). IHS describes retinal migraine as “Repeated attacks of monocular visual disturbance, including scintillations, scotomata, or blindness, associated with migraine headache” and provides diagnostic criteria for this disorder. Therefore, retinal migraine can be considered both an ophthalmic and a neurologic disorder. Although migraine in general is one of the most prevalent causes of disability (second among young women worldwide), retinal migraine is rare. Still, its complex pathogenesis may lead to incomplete diagnoses, and its actual prevalence may be higher than reported, though it remains unknown. In some migraine cases, headaches are preceded by symptoms called aura or migraine aura (4). The most common type of aura is a visual aura, experienced by more than 90% of patients who have migraine with aura (5). Migraine with visual aura is occasionally referred to as “ocular migraine” or “ophthalmic migraine”; however, these terms will not be used in this paper since they also refer to retinal migraine.

AMD is the leading cause of legal blindness and vision loss among older adults worldwide. In its advanced form, AMD causes severe disturbances in central vision, similar to those experienced during a visual aura. Clinically, it manifests in two forms: dry and wet (neovascular). Both forms are incurable; however, the latter can be treated with antibodies against vascular endothelial growth factor A (VEGFA) and its receptor. This treatment for wet AMD slows disease progression and delays sight loss (6). AMD has been linked to various conditions, such as multiple systemic diseases, cognitive decline, and mental and social health issues (7). A 2022 study presents population-based evidence that persons with migraine have 20% higher risk of subsequently being diagnosed with neovascular AMD (8). Shared mechanisms in AMD and migraine, such as microvascular dysfunction or altered cellular stress responses, have been proposed but require further validation.

Transient receptor potential (TRP) channels are expressed in trigeminal neurons and brain regions crucial for migraine pathogenesis, converting noxious stimuli into pain signals (9). Their functional role in migraine has been suggested in several studies, making them a therapeutic target for migraine [reviewed in (10)]. TRP channels are signaling molecules, and defects in their genes are associated with many diseases, including hereditary disorders, TRP channelopathies (11). Most of TRP channels are accessible to drugs as they are located on the cell surface. As TRP channels are multifunctional, they pose a challenge as therapeutic targets due to the need to minimize potential side effects. Their role in retinal physiology and migraine remains an area of ongoing research (12). While some migraine therapies may influence TRP-mediated pathways, their precise contribution to treatment effects is not fully understood (13). Additional clinical observations suggest that photophobia, retinal vascular events, and changes in retinal nerve fiber layer thickness may occur more frequently in individuals with migraine than in the general population (6, 14). However, causal relationships and underlying mechanisms remain unclear, and these associations should be interpreted cautiously.

Our search strategy focused on potential associations between migraine and retinal disorders, focusing on retinal migraine, AMD, retinal vascular occlusions, and photophobia. The search drew on publications from PubMed, Embase, Google Scholar, ScienceDirect, and the Cochrane Library. We used search terms combining “migraine,” “retina,” “AMD,” “photophobia,” “vascular occlusion,” and “retinal nerve fiber” in various configurations. Publications from the past 10 years were prioritized unless earlier foundational studies were essential. The scope included human studies and relevant animal models. All article types, including original research, reviews, and meta-analyses, were included, with no language restrictions. We did not apply any parameters to select the most critical articles. Instead, we evaluated each based on essential content. As a narrative review, this work reflects an interpretive synthesis rather than a systematic analysis.

## 2 Retinal homeostasis

The human neural retina (neuroretina) is a peripheral component of the central nervous system (CNS), derived from the forebrain, and represents the only CNS structure that can be visualized noninvasively. It originates from the neural ectoderm of the optic cup, itself formed as an evagination of the diencephalon, and consists of interconnected neuronal and supporting cell types (15).

**Figure 1:**
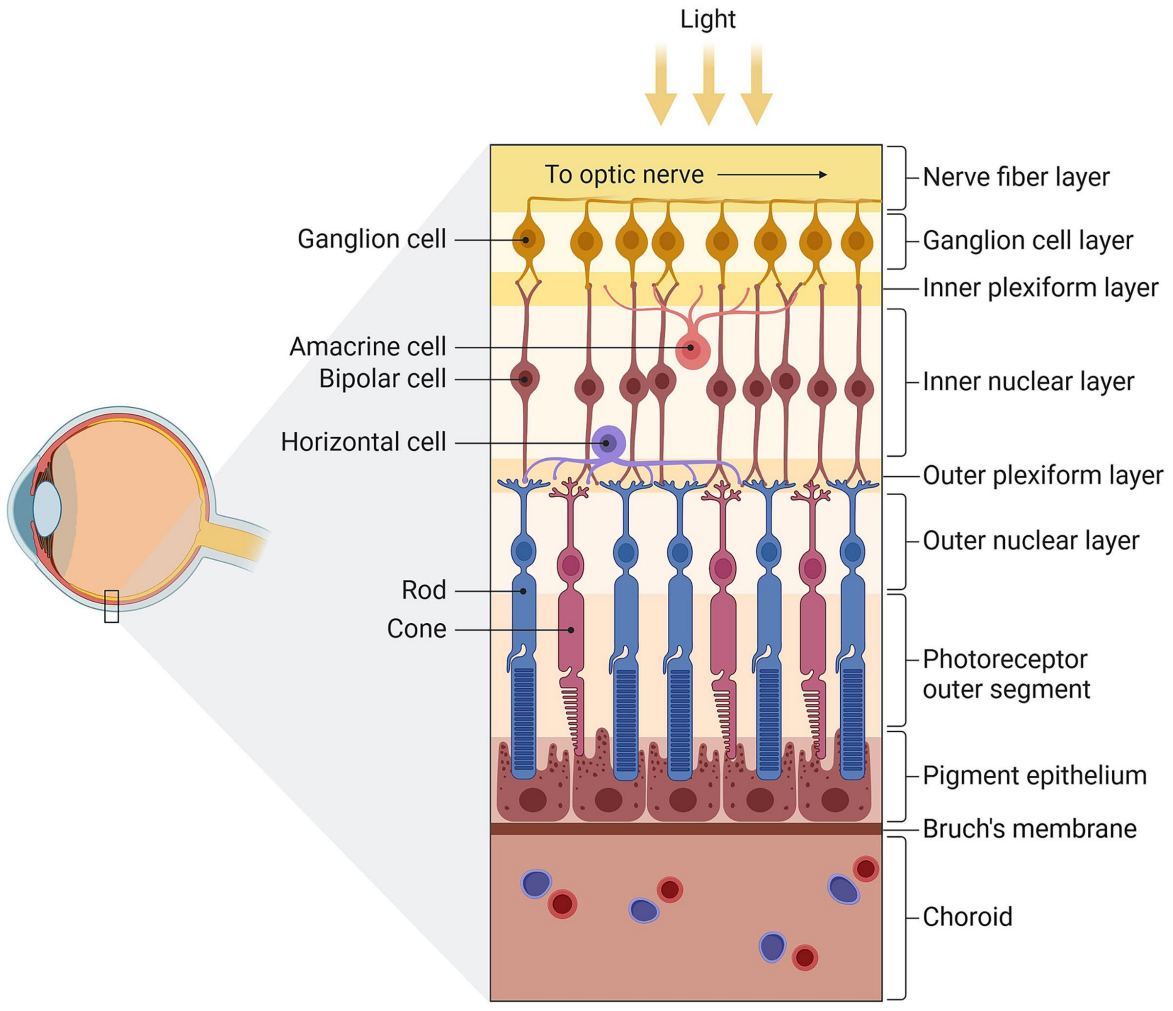
Schematic representation of the human retina. Created in BioRender. Błasiak, J. (2026) https://BioRender.com/ppicrpg.

Structurally, the neuroretina is organized into multiple layers (Figure 1). The innermost layer contains retinal ganglion cells, whose axons form the optic nerve, while the outermost layer comprises rod and cone photoreceptors. Between these layers lie the inner and outer nuclear layers, which contain the cell bodies of interneurons and photoreceptors, respectively. The inner nuclear layer includes bipolar, horizontal, and amacrine cells that mediate signal transmission from photoreceptors to ganglion cells. Synaptic interactions occur within the outer plexiform layer (between photoreceptors and bipolar cells) and the inner plexiform layer (between bipolar, amacrine, and ganglion cells). Photoreceptor outer segments interface with the retinal pigment epithelium (RPE), which is separated from the choroid by Bruch’s membrane. In addition to supporting photoreceptor function, the RPE contributes to retinal homeostasis through nutrient transport, ion balance, and phagocytosis of photoreceptor outer segments, and it is implicated in the pathogenesis of several retinal diseases, including AMD. Retinal homeostasis is vital for maintaining normal visual functions. Essential components of retinal homeostasis include its integrity, the integrity of the retina-blood barrier, an efficient immune-inflammatory response, and metabolic regulation. Redox balance is a critical aspect of retinal homeostasis.

Energy metabolism is essential for retinal homeostasis for two main reasons (16). First, the brain, which accounts for about 2% of body weight, is one of the most energy-demanding tissues in the human body, consuming approximately 20% of total oxygen and around 25% of total glucose for energy (17). The visual system, in turn, is among the brain’s highest-energy consumers (18). Deficiency in energy metabolism can result in visual impairments and blindness (16). Second, energy is generated by oxidative phosphorylation (OXPHOS) in mitochondria, the primary energy-generating pathway in neurons, or by glycolysis in the cytosol, favored by glial cells. Oxygen for OXPHOS is supplied by blood, making the retina one of the tissues with the highest blood flow. This intense oxygen metabolism produces reactive oxygen and nitrogen species (RONS), even under normal conditions. RONS production increases when OXPHOS is impaired. These RONS may contribute to oxidative stress and damage cellular macromolecules, leading to degenerative changes in the retina, as seen in retinal degenerative diseases such as AMD, retinitis pigmentosa, and diabetic retinopathy. Therefore, understanding how retinal cells manage excess RONS, or, more broadly, oxidative stress, is crucial. Cells use three main antioxidant defense pathways, including (i) antioxidant enzymes that neutralize (scavenge) RONS; (ii) DNA repair proteins that fix oxidative DNA damage; and (iii) low-molecular-weight antioxidants, including certain vitamins and peptides that deactivate RONS. However, the importance of the latter pathway is relatively low compared to the first two, even though it is the easiest to enhance through dietary supplementation.

Although it is challenging to identify a disease without oxidative stress implicated in its pathogenesis, the intensity of oxidative events in the retina makes it particularly susceptible to oxidative-stress-related disorders. Specifically, oxidative stress is frequently implicated in AMD pathogenesis (19).

## 3 Migraine

Although migraine is one of the most disabling syndromes, its mechanisms of pathogenesis remain not fully understood. This is primarily due to challenges in studying relevant human material, even with advanced neuroimaging techniques, and to inherent limitations of using animal models to study human migraine. These limitations are mainly associated with the fact that the diagnosis of migraine is often confined to self-reports.

Migraine was once considered a vascular disorder due to meningeal vasodilation during its course. Today, migraine is acknowledged as a complex neurological disease, with its etiology involving various cortical, subcortical, and brainstem regions that regulate autonomic, affective, cognitive, and sensory functions (12). However, the mechanisms that activate these regions and the progression leading to migraines remain not entirely understood.

Despite these challenges, understanding the role of calcitonin gene-related peptide (CGRP) in the pathogenesis of migraine and targeting CGRP and its receptor in migraine therapy have revolutionized the treatment of this condition (20). The next milestone in understanding migraine pathogenesis was the discovery of another neuropeptide, pituitary cyclase-activating polypeptide (PACAP), which, along with its receptor, has emerged as a therapeutic target (20). Both CGRP and PACAP may trigger delayed migraine-like headaches in migraine patients, but they tend to cause only mild immediate headaches in some control subjects [reviewed in (21)]. These results indicate that CGRP and PACAP are not solely responsible for the acquisition of the migraine phenotype. However, preclinical studies aimed at understanding how CGRP may trigger migraine attacks are valuable for the overall understanding of migraine pathogenesis.

**Figure 2:**
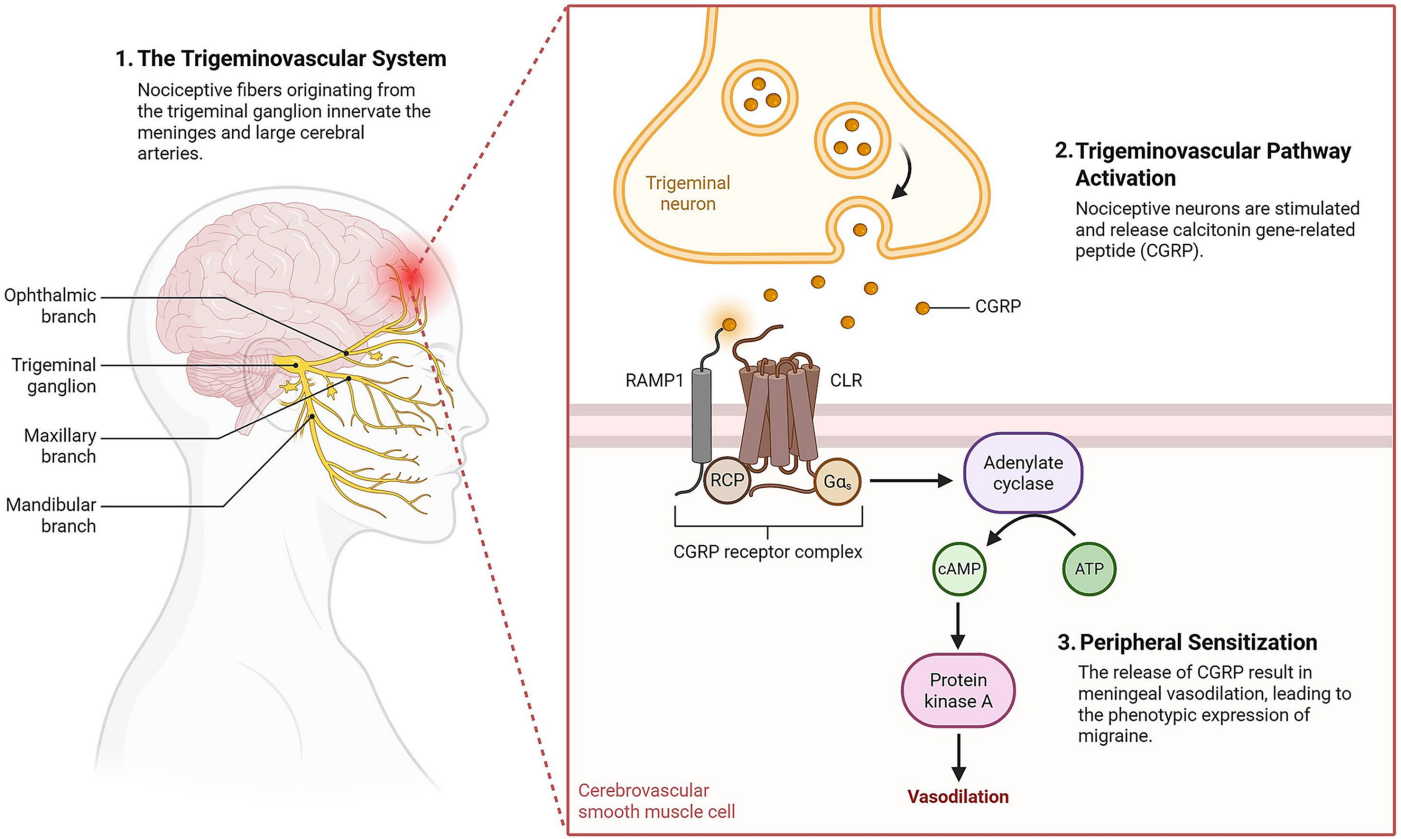
The trigeminovascular system and calcitonin gene-related peptide (CGRP) play significant roles in migraine. The trigeminovascular system is a network of neurons originating in the trigeminal ganglion and innervating the cerebral vasculature, including the dura mater. Nociceptive fibers innervate the meninges and large cerebral arteries. When stimulated, nociceptive neurons release CGRP, which causes meningeal vasodilation and leads to migraine symptoms. The CGRP receptor complex consists of a calcitonin receptor-like receptor (CLR), receptor activity-modifying protein 1 (RAMP1), and two cytoplasmic proteins: a G protein containing the Gsα subunit (Gαs), which stimulates adenylyl cyclase and the cAMP-dependent signaling pathways, and receptor-coupling protein (RCP). Created in BioRender. Blasiak, J. (2026) https://BioRender.com/37y3yx7.

The trigeminovascular system plays a role in pain transmission during a migraine attack, as indicated by the release of CGRP (13) (Figure 2). When nerves are stimulated, CGRP is released from its storage vesicles through calcium-mediated exocytosis. The release of CGRP is regulated by presynaptic receptors on trigeminal neurons. In bipolar trigeminal sensory neurons located in the trigeminal ganglion, CGRP is released at both peripheral and central nerve terminals. The functional CGRP receptor is a heterodimer comprising a seven-transmembrane, G protein-coupled calcitonin receptor-like receptor (CLR) and a single membrane-spanning receptor activity-modifying protein 1 (RAMP1) (22). This transmembrane CGRP receptor complex also includes two cytoplasmic proteins that are linked to the CLR-RAMP1 heterodimer to facilitate signal transduction. CLR is associated with a G-protein containing the Gsα subunit (Gαs), which stimulates adenylyl cyclase and activates the cAMP-dependent signaling pathways. RAMP1 and CLR are associated with another cytoplasmic protein, receptor coupling protein (RCP), which enhances G-protein stimulation (23). A CGRP-mediated increase in intracellular cAMP activates protein kinase A, which phosphorylates various downstream targets and induces vasodilation in cerebrovascular smooth muscle. Activating the CGRP receptor may play a role in neurogenic inflammation and in sensitizing neurons in the nociceptive pathway (24). Therefore, CGRP may play a causal role in migraine pathophysiology, but it is not the only biomolecule contributing to it.

Migraine can be associated with sensory disturbances, most commonly visual or auditory, referred to as aura, which may occur before or simultaneously with migraine headaches (25). Cortical spreading depolarization (CSD) refers to a widespread loss of ion homeostasis, altered vascular responses, changes in synaptic architecture, and a subsequent decrease in cortical electrical activity (26). CSD may activate central trigeminovascular neurons and trigger CGRP expression (27, 28). The role of CSD in migraine with aura is a topic of debate. CSD is said to underlie migraine aura, including its positive symptoms like mosaic patterns and negative symptoms such as scotoma, along with a similar propagation speed and vasoreaction pattern (29). However, some reports question these claims (30).

## 4 Retinal migraine

Retinal migraine is defined by monocular vision loss occurring alongside migraine headaches. According to the IHS ICHD-3 criteria, both vision loss and other visual disturbances, such as scotoma and scintillation, should be fully reversible and typically last less than 1 h. However, this criterion is questioned is in question, as some studies indicate that irreversible vision loss can be part of the retinal migraine spectrum (31). Isolated case reports have also described retinal or optic nerve ischemic events in patients with presumed retinal migraine, although such observations remain limited and difficult to interpret (32).

Retinal migraine is considered a rare entity, and its true prevalence is uncertain, in part due to evolving diagnostic criteria and challenges in distinguishing it from other causes of transient monocular vision loss [reviewed in (33)]. A 2021 study identified only 12 cases of the condition following the ICH-3 criteria over a 15-year period, underscoring the limited number of well-characterized cases available for study (34). This rarity, combined with the need to exclude alternative diagnoses, such as amaurosis fugax, carotid artery disease, or inflammatory vascular conditions, further complicates its clinical characterization (33).

Given that cases of retinal migraine are exceedingly rare, it is unsurprising that the underlying pathophysiology of this condition remains largely unknown. Several key characteristics of retinal migraine, including transient monocular visual loss lasting less than 1 h, prolonged transient monocular visual loss, and permanent transient monocular visual loss, correspond clinically to the typical visual aura of migraine, prolonged aura, and migrainous infarction, respectively (31). These three effects might share common mechanisms in the brain and the retina. However, these comparisons are largely speculative.

Since CSD is said to be linked with migraine with aura and has been observed in the chicken retina, similar effects probably occur in the retinas of patients with retinal migraines (35). One patient with retinal migraines described her vision disturbance as black paint slowly moving across her visual field, which could result from CSD in the retina (36). Observations from animal models and individual case descriptions are insufficient to establish a consistent mechanistic framework.

Primary vascular dysregulation associated with retinal vascular disease, which occurs alongside migraines, is believed to contribute to the pathogenesis of retinal migraines (31, 37). Ischemia is often cited as a cause of permanent monocular visual loss in the context of migraine; therefore, it can be considered a potential factor in the pathogenesis of retinal migraine.

Overall, retinal migraine remains a rare and somewhat controversial clinical diagnosis, with limited epidemiological confirmation and ongoing debate regarding its diagnostic boundaries. Although it is recognized in ICHD-3, the available evidence is largely based on case reports and small series, which constrains the ability to draw robust or generalizable mechanistic conclusions. In particular, distinguishing true retinal phenomena from more common cortical visual aura can be challenging, raising concerns about potential misdiagnosis. Given these limitations, retinal migraine should be interpreted with caution and not be considered a primary biological model for understanding broader retina-migraine interactions. Instead, it may represent a distinct and insufficiently characterized entity whose relevance to general migraine pathophysiology remains to be clarified through more systematic investigation.

## 5 Age-related macular degeneration

Age-related macular degeneration is an eye disease whose incidence increases significantly with age, posing an emerging problem for the healthcare system as populations age. It affects the macula, a small area at the center of the retina that contains the fovea, which is responsible for central, color, and high-resolution vision. This condition can lead to severe vision impairment and loss.

**Figure 3:**
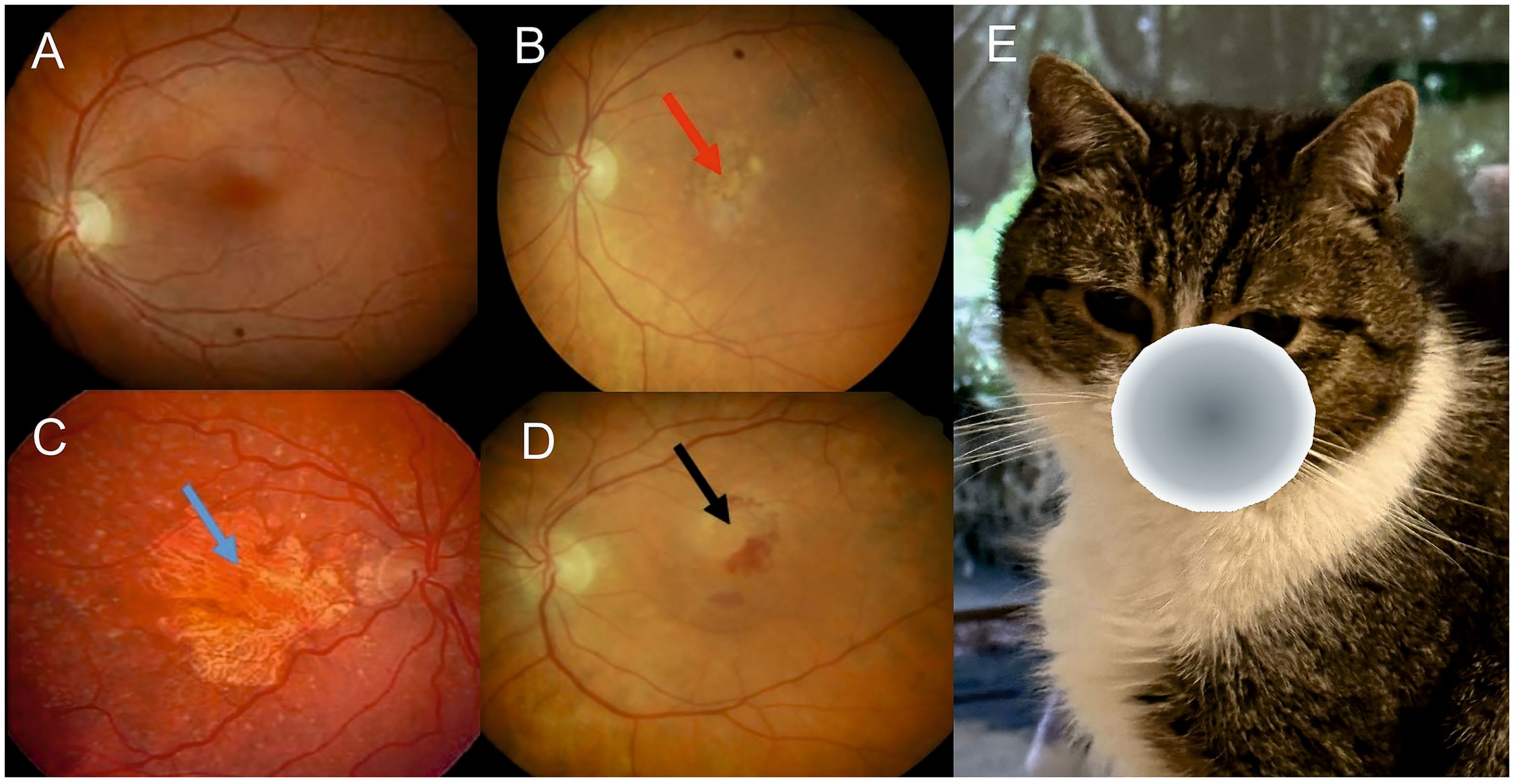
Age-related macular degeneration (AMD) is illustrated with fundus images, showing the healthy macula **(A)**, the macula affected by intermediate dry AMD **(B)**, late dry AMD characterized by geographic atrophy **(C)**, and neovascular AMD **(D)**. The red arrow points to the accumulation of drusen, the blue arrow indicates geographic atrophy, and the black arrow highlights hemorrhages and edema. In neovascular AMD, subretinal hemorrhage causes disturbances or loss of central vision, symbolically represented in **(E)**. The fundus pictures were taken by Professor K. Kaarniranta.

The early stage of AMD is marked by pigmentation abnormalities in the retinal pigment epithelium (RPE) and the presence of drusen, which are extracellular structures made up of lipids and proteins (38). In its advanced stage, AMD manifests in two clinically distinguishable forms: dry (non-exudative, atrophic) and neovascular (exudative, neovascular) AMD (Figure 3). Dry AMD is the initial form of the disease that may progress to neovascular AMD, accounting for 15–20% of all advanced AMD cases (39). The late stage of dry AMD, known as geographic atrophy (GA), is associated with the death of photoreceptors and the atrophy of the supporting RPE cells and the choriocapillaris, and may lead to vision loss (40). Despite numerous preclinical studies and several clinical trials, advanced dry AMD remains untreatable. AMD is a complex disease with numerous potential risk factors, and the interaction among them may contribute to AMD pathogenesis. These factors include genetic, epigenetic, environmental, and lifestyle elements, many of which are related to oxidative stress. Furthermore, the retina exhibits high oxygen metabolism, leading to excessive production of reactive oxygen and nitrogen species (RONS), which can damage cellular components. Additionally, aging, regarded as the primary risk factor for AMD, is also linked to oxidative stress (41). Although several pathways link oxidative stress to AMD pathogenesis, it remains unclear whether this stress is a causative factor, a consequence of AMD, or both (42). Despite well-established diagnostic criteria and procedures, along with a clear clinical picture of AMD, it remains an undertreated condition. The main reason for this is the incomplete understanding of the mechanisms behind AMD pathogenesis. This situation is further complicated by slow progress in experimental studies of the disease’s molecular basis, hindered by limited access to human target material and by limitations in animal models of human AMD (43). As a result, there are no AMD-specific preventive recommendations available in Europe (44). However, the introduction of medications that target vascular endothelial growth factor A (VEGFA) and its receptor has been a breakthrough in treating AMD, but in some instances of neovascular AMD, anti-VEGFA treatment is found to be ineffective, and the reasons for this remain largely unknown (45).

A large-scale retrospective study involving a Taiwanese population, which included 20,333 neovascular AMD patients and 81,332 controls, found that migraine patients had a 20% higher risk of neovascular AMD compared to the controls (8).

Several factors may potentially contribute to the connection between migraine and AMD, with oxidative stress related being one of the primary candidates. RONS are produced in the energy-deficient brains of migraine patients, requiring significant energy production linked to the excess of RONS (46). Furthermore, many migraine triggers lead to oxidative stress and an increase in RONS production (47). Transient receptor potential cation channel subfamily A member 1 (TRPA1), part of the TRP channels superfamily, is activated by various migraine triggers and is expressed in trigeminal neurons and brain regions crucial to migraine pathogenesis (48). It translates oxidative stress and triggers neurogenic inflammation, suggesting that oxidative stress could act as the common factor connecting migraine triggers (47).

We showed that AMD patients had a higher level of basal and oxidative endogenous DNA damage than controls (49). Conversely, we suggested that the DNA damage response (DDR) might be impaired in migraine (50). Compromised DDR in migraine may be evident in DNA single-strand break repair, as it has been reported to play an important role in CNS neuronal function and, consequently, in the nervous system phenotype (51, 52).

Autophagy, a process that removes damaged, dysfunctional, or unnecessary cellular components, is strongly implicated in AMD pathogenesis (53). Conversely, we posited that autophagy could protect the migraine-affected brain from the prolonged consequences of headache attacks (54). However, this is still in the hypothesis phase and needs verification by experimental/clinical data.

The challenge in exploring the shared aspects of migraine and AMD pathogenesis is that AMD, as its name implies, is an age-related disease, whereas migraine is not. However, determining the timing of AMD onset, which may be triggered by vascular changes presenting as disease phenotypes in later life, is generally impossible. Given the roles of vascular and immune factors in the pathogenesis of AMD and migraine, neovascular AMD is more likely to be associated with migraine than its dry counterpart.

**Figure 4:**
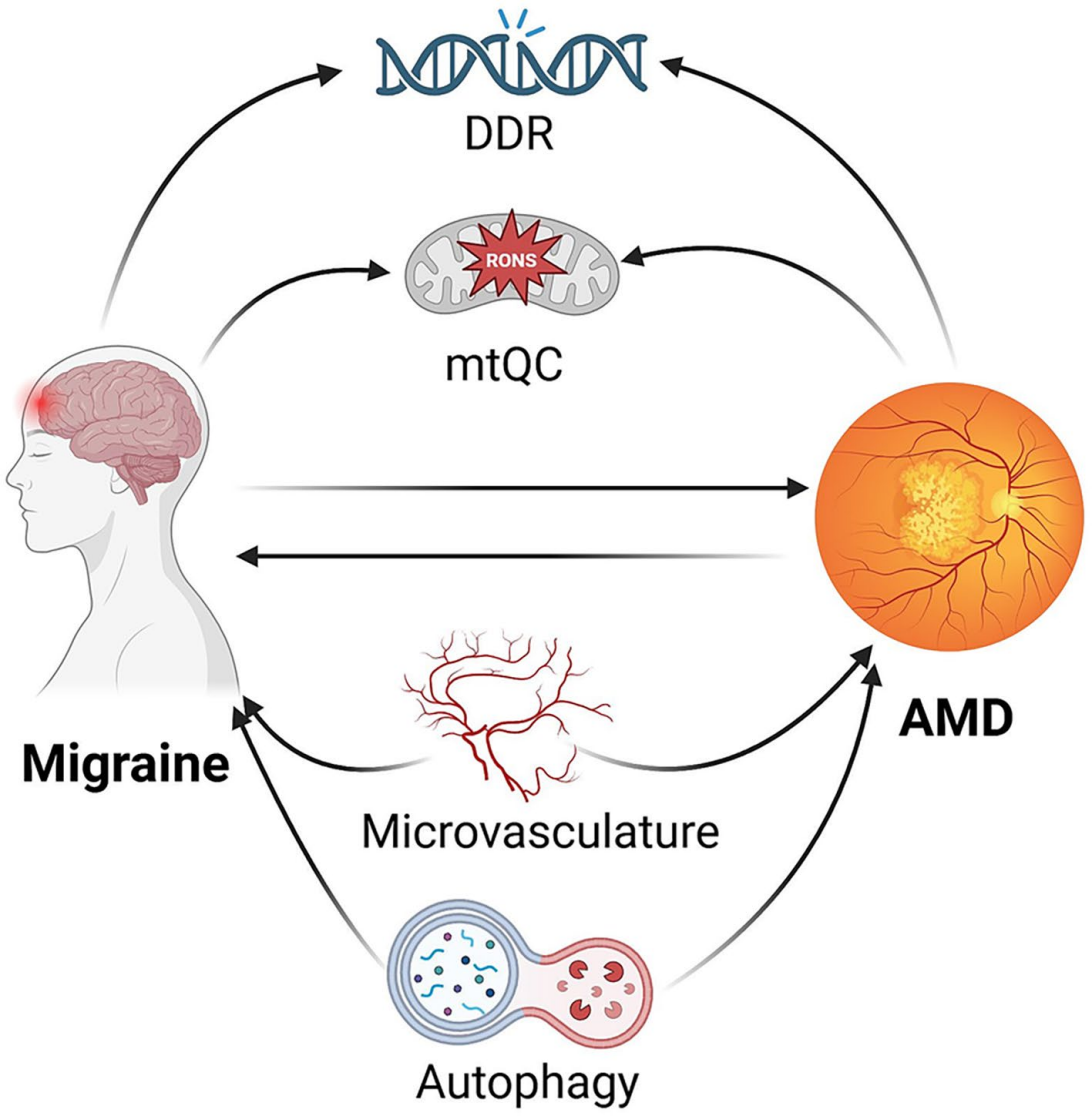
Age-related macular degeneration (AMD), depicted here as an AMD-affected fundus, has been hypothesized to share specific aspects of pathogenesis with migraine, which may lead to an increased risk of AMD among migraine patients and vice versa. The key features of this hypothetically shared pathogenesis include a compromised DNA damage response, as indicated by DNA single-strand breaks, impaired mitochondrial quality control (mtQC) resulting in the overproduction of reactive oxygen and nitrogen species (RONS), microvascular changes, and alterations in autophagy. Created in BioRender. Błasiak, J. (2026) https://BioRender.com/idt1bxf.

In summary, several pathogenic mechanisms may hypothetically associate with both migraine and AMD, including DDR, autophagy, and mtQC (Figure 4). These mechanisms may be disrupted by oxidative stress and could also contribute to other symptoms with similar pathogenesis to migraine, which will be addressed in subsequent sections.

## 6 Retinal vascular occlusion

Retinal vascular occlusions can lead to visual impairments, including vision loss, and are categorized into two types: retinal artery occlusions (RAOs) and retinal vein occlusions (RVOs) (55). While RAOs and RVOs can be classified as syndromes, there are significant differences in their pathogenesis, systemic implications, and management. Both RAOs and RVOs are more common among the elderly and are closely linked to cardiovascular risk (56). However, RVOs typically do not require systemic intervention. In contrast, RAOs, which include vascular transient monocular vision, branch retinal arterial occlusion, central retinal arterial occlusion, and ophthalmic arterial occlusion, are linked to a higher risk of stroke and cardiac events that must be addressed aggressively (57). The pathogenesis of both syndromes remains largely unknown, but it has been suggested that RVOs may be underpinned by vessel damage, stasis, and hypercoagulability– the Virchow’s triad for thrombogenesis (58). Thrombogenesis results from atherosclerosis of the retinal artery, leading to compression of the retinal vein in the lamina cribrosa and subsequent thrombosis (59).

Given that cardiovascular disorders, including ischemic and hemorrhagic strokes, are more prevalent among migraine patients compared to the general population, it is reasonable to hypothesize that these individuals may face an increased risk of retinal artery occlusion (59).

A 2024 population-based retrospective cohort study involving 620,760 migraine patients and an equal number of controls found that migraine patients had higher cumulative incidences of subsequent retinal vascular occlusion, including both RAO and RVO (60). A higher risk ratio for retinal artery occlusion was observed in migraine with and without aura. Additionally, the use of nonsteroidal anti-inflammatory drugs, propranolol, and flunarizine was found to reduce the risks of retinal vascular occlusion. This study was conducted entirely within a Taiwanese population; however, retinal vascular occlusion has been reported to vary by race and ethnicity (61). A US study reported a threefold increase in the risk of RAO in American migraine patients, while the Taiwanese study indicated a twofold increase (14). This difference may be partly explained by variations in ethnicity and race, such as the higher prevalence of giant cell arteritis in Caucasians compared to Asians. However, all these studies suffer from incomplete characterization of individuals and the population enrolled in the study. Whereas “insurance claims database,” as in paper (14) may be useful for drawing epidemiological conclusions, it is not enough to determine the potential link between RAO and migraine. Also, “American migraine patients” may be represented by significantly different phenotypes, some of which may be confounding factors of the analysis.

The incidence of RAOs is greater in men than in women and rises with age (62). In the cited Taiwanese study, migraine patients aged 20–40 years had a higher risk of retinal vascular occlusion than those aged 40–60 or 60–80 years (60). These results align with the general trend of migraine and arterial disease prevalence with age, including large artery atherosclerosis and atrial fibrillation. Retinal vascular occlusion, particularly central retinal artery occlusion, which is typically associated with sudden, painless vision loss in one eye, significantly increases the risk of brain stroke. Therefore, patients diagnosed with central retinal artery occlusion require urgent medical attention (63). On the other hand, stroke prevalence is higher among migraine patients; thus, the triad of RAO, migraine, and stroke might serve as evidence of the significance of retinal mechanisms in CNS diseases, including migraine (63).

In summary, current evidence on a potential association between RAO and migraine is mainly from large-cohort studies, which generally are characterized by inter-study inconsistency and lack the exact characteristics of migraine patients. Some studies, including those on the ictal and interictal phases of migraine in connection with RAO, are challenging and should be addressed first in animal or in-vitro models.

## 7 Photophobia

Photophobia is often linked to migraine and may be a part of the migraine aura (64). It can occur both during and between headache attacks, and it may be triggered by retinal stimulation from bright, flickering, or patterned light with specific colors (7). Although photophobia in migraine has historically been attributed to cortical impairments, the discovery of intrinsically photosensitive retinal ganglion cells (ipRGCs) led to studies connecting migraine photophobia with retinal phenomena (65, 66). The primary input to the ipRGCs comes from rod and cone photoreceptors, and the ipRGCs may regulate the output of photoreceptors through amacrine cell activity (67, 68).

Like the general pathogenesis of migraine, the trigeminovascular system may play a role in migraine photophobia. The trigeminal ganglion projects to the trigeminal nucleus caudalis, from which second-order neurons send signals to the posterior thalamic area (PTA), containing CGRP receptors (69). Research involving experimental animals suggests that the PTA might integrate light and pain signals, which are directly related to migraine photophobia (12, 70).

Migraine patients reported various changes in headache severity with different light intensities and colors (71). During the ictal phase, white, blue, amber, and red lights aggravated headaches in approximately 80% of patients, while in the interictal phase, light-induced headaches occurred in only 16–19% of cases. Green light intensified headaches in 40% of patients during the ictal phase and triggered headaches in 3% during the interictal phase. Essentially, no headaches in response to light stimulation were reported by control subjects. Therefore, color preference in photophobia is a distinctive characteristic of migraine, regardless of its phase.

The involvement of retinal mechanisms in photophobia is supported by the observation that it can occur even in blind patients who cannot detect light due to the removal of a pituitary adenoma or who have functionally inactive rods and cones (72). Further studies conducted on 20 individuals confirmed the involvement of ipRGCs in photophobia overall and specifically in migraine (70). Additionally, there is an indirect pathway between the optic nerve and the trigeminal nerve, as in ipRGCs and in subcortical structures, including the basal ganglia, thalamus, and hypothalamus (73–75).

It may be hypothesized that different mechanisms drive migraine photophobia than those found in ocular disorders (14). However, studies aimed at identifying which retinal cells underlie migraine photophobia have been inconsistent. The question of whether migraine photophobia is solely underlined by retinal mechanisms remains unresolved, particularly since some cases of migraine photophobia are better explained by cortical mechanisms (14).

In summary, migraine photophobia may be influenced by both cortical and retinal mechanisms, but their contributions may vary depending on the phase of the disease. The cortical mechanism is likely determined during the interictal phase, but it may be insufficient during the ictal phase, when the cortex is affected by migraine-related phenomena. Therefore, further studies are needed, preferably using the electroretinal response and comparing it with its electroencephalographic counterpart in migraine patients during headache attacks, as such studies are currently scarce.

## 8 Retinal nerve fiber layer thickness

The retinal nerve fiber layer (RNFL) is part of the retina, primarily composed of ganglion cell axons, which are covered by astrocytes and grouped by Müller cell extensions (76). The pain-inducing neuropeptides released by the activated trigeminovascular system, such as CGRP and PACAP, lead to abnormal vasoconstriction and alterations in perfusion pressure (77). These changes are believed to be prolonged due to the nature of migraines, which affect both the brain and the retina, ultimately leading to ganglion cell death (78, 79). Consequently, the RNFL, primarily composed of ganglion cells, is a potential target of these changes.

**Figure 5:**
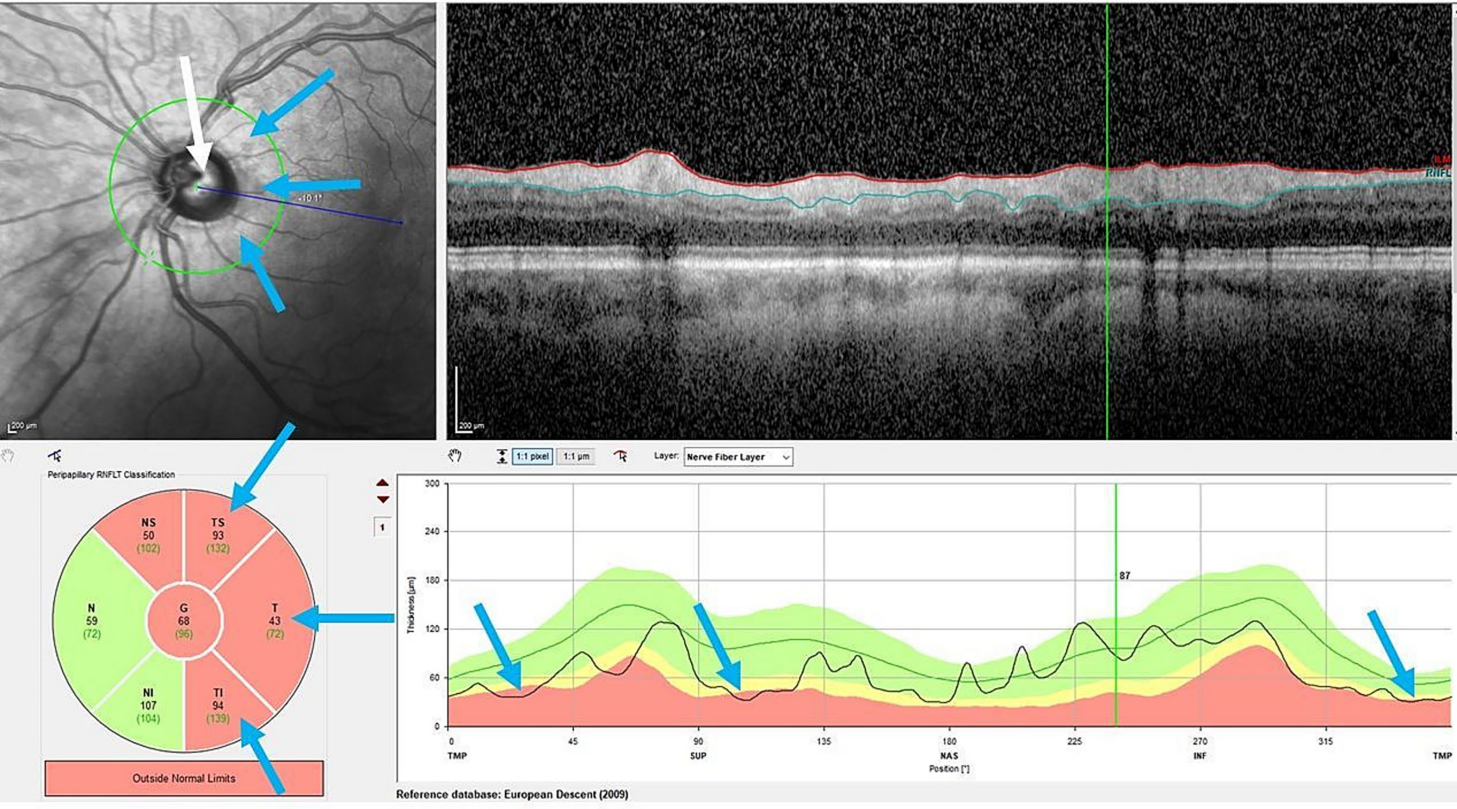
Optical coherent tomography (OCT) image from the optic disc area of a normal tension glaucoma case shows decreased thickness of the retinal nerve fiber layer (RNFL). Abbreviations: G, optic disc; T, temporal; TS, temporal superior; NS, nasal superior; N, nasal; NI, nasal inferior; TMP, temporal; SUP, superior; NAS, nasal; INF, inferior. Retinal thickness values are in micrometers, and reference values are shown in brackets. White arrows indicate pathological optic disc excavation, while blue arrows show decreased RNFLs. The green color represents normal states, and red indicates pathological conditions. The OCT images were taken by Professor K. Kaarniranta.

The primary method for studying RNFL is optical coherence tomography (OCT) and its variants; however, certain methodological issues must be addressed in OCT-based studies, as these may reveal inconsistencies in the results and interpretation of RNFL thickness measurements, particularly since time-domain (TD-OCT) and spectral-domain (SD-OCT) show significant differences in sample preparation (79). Figure 5 presents typical RNFL measurement results in normal-tension glaucoma.

A 2023 meta-analysis found reduced RNFL thickness in migraine patients compared to controls (79). The decrease in RNFL thickness was more pronounced in the superior and inferior quadrants of the four-zone division among migraine patients with aura, compared to those without aura. In terms of technique, SD-OCT performed better than TD-OCT in measuring the reduction of RNFL thickness. Decreased RNFL can be observed in glaucoma. It is important to recognize that migraine attacks may interfere with standard glaucoma tests, including visual fields, electrophysiology, and ocular imaging. The possible connection between migraine and glaucoma is further complicated by epidemiological data suggesting an increased prevalence of migraine in patients with glaucoma, particularly those with normal tension glaucoma (80).

The reasons for the reduction in RNFL thickness are unclear, but several hypotheses have been proposed. It is important to note that this effect may indicate structural changes in the CNS associated with migraine, which remains a topic of debate. The simplest hypothesis suggests that the decrease in RNFL thickness may be linked to vascular abnormalities, such as vascular dysregulation (vasospastic diathesis) or focal cerebral ischemia (81). This hypothesis was supported by study findings indicating reduced blood flow in the central retinal artery and posterior ciliary artery in migraine patients, as assessed using color Doppler ultrasound to evaluate retinal vascular and perfusion changes (82). Changes in the blood supply to the optic nerve and RNFL may cause hypoxic injury, leading to the death of retinal ganglion cells, which are associated with underlying retinal vascular diseases in patients with migraines (83, 84).

The fundamental question is how specific the changes in retinal nerve fiber layer thickness are to migraine. Several studies suggest that RNFL thickness changes occur with normal tension glaucoma, but the glaucoma patients with migraine exhibit greater changes than those without migraine (85, 86). Additionally, RNFL changes are observed in multiple sclerosis. Consequently, since changes in RNFL thickness may be associated with various symptoms, it is difficult to assess how much they can be primarily attributed to migraine. Therefore, RNFL changes cannot be considered a relatively stable retinal biomarker of migraine; they are promising but not yet definitive.

## 9 Conclusions, perspectives and outstanding questions

The retina is a part of the CNS and may be directly affected by CNS disorders. Conversely, retinal diseases that manifest as visual disturbances may lead to changes in CNS function. This interplay between the retina and CNS disorders can be significant for their diagnosis, prevention, and treatment. Migraine, a CNS condition, is often linked with visual disturbances. Thus, it is crucial to determine whether and how these disturbances are specific to the retina. Additionally, some ophthalmic retinal-related syndromes are reported more frequently in migraine patients than in the general population. However, a significant part of these studies suffers from methodological inconsistencies, does not provide all the necessary information, and in general, their results remain uncertain.

In this review, we demonstrated that some symptoms primarily regarded as ophthalmic disorders, such as retinal migraine, AMD, photophobia, retinal vascular occlusions, and changes in retinal nerve layer thickness, were reported to occur more frequently in migraine patients than in healthy individuals. This may represent an association and shared vulnerability rather than migraine-specific retinal pathogenic mechanisms. As other population studies on migraine-retinal connection, they lack clear inclusion and exclusion criteria, complete characterization of the population and general standardization.

Retinal migraine appears to be the most direct link between migraine and the retina. However, the number of cases identified so far is too small to present any results as significant (87). Most recent papers on retinal migraine consist of case reports and reviews (32, 33). Considering studies that report permanent vision loss in patients with retinal migraine, it should be determined whether this loss is a complication associated with retinal migraine or rather an inherent characteristic of an advanced form of the disease. Therefore, redefining retinal migraine might be necessary. The extent to which the incidence of permanent loss of visual acuity or visual field related to retinal migraine could be underreported and/or underestimated is significant (88).

Given that cortical mechanisms contribute to photophobia, further investigation is needed to explore the contribution of retinal mechanisms to photophobia in migraine, particularly during its ictal phase, as this may illuminate the relationship between the cortex and the retina in migraine.

There are several shared pathogenic mechanisms in AMD, largely linked to oxidative stress, a common factor in various diseases. However, unlike retinal migraines and some other syndromes related to migraine, reports suggest that impaired DDR, autophagy, vascular homeostasis, and mitochondria may be elements significant for migraine and AMD pathogenesis. A closer examination of patients with comorbid migraine and AMD may enhance our understanding of the pathogeneses of both conditions. It should be noted that the correlation between migraine and AMD pertains specifically to the neovascular form of AMD; no studies have investigated dry AMD in this context. The differences between dry and neovascular AMD necessitate significantly different management strategies, making it crucial to explore a correlation between migraine and dry AMD (89).

AMD and retinal vascular occlusion exhibit age and sex dependencies that are not aligned with those seen in migraine. However, these diseases are considered during their clinically detectable phases, making it challenging, if not impossible, to ascertain a molecular history of the symptoms.

Another symptom that may connect migraine with the retina is diabetic retinopathy (DR), a significant complication of diabetes mellitus characterized by the growth of abnormal blood vessels in the retina (90). We have not analyzed the connection between migraine and DR for two reasons. First, diabetes mellitus, particularly type 2, affects numerous organs and functions in the human body, making it challenging to identify a correlation between just two parameters. Second, current research suggests that diabetes may protect against migraine (91–93). A 2022 nationwide Danish cross-sectional study involving over 1.2 million people found that patients with diabetic retinopathy (DR) had a lower risk of prevalent migraine, but DR was not a protective factor against new-onset migraines (94). Therefore, the relationship between migraine and DR should be further investigated to explore their common aspects of pathogenesis.

The final question is whether the retina is directly affected by, or involved in, the electrophysiological phenomena that occur during the initiation and progression of migraine headaches. What is the role of the RPE in these phenomena? It is too soon to answer these questions, which should be addressed in further studies.

This review has several limitations that should be considered when interpreting its conclusions. First, the included studies show substantial heterogeneity in design, populations, diagnostic criteria, and assessed outcomes, which limits comparability and precludes a firm synthesis of findings. As a narrative review, this work is inherently subject to selection bias and does not follow systematic review methodology, including predefined inclusion criteria, quality assessment, or quantitative analysis. The evidence base itself is variable, with considerable differences in sample sizes, ranging from small clinical series to larger observational datasets, each with distinct sources of bias. Many of the reported associations rely on retrospective or cross-sectional studies, which are particularly vulnerable to confounding factors such as age, vascular comorbidities, and lifestyle influences, as well as potential ascertainment and diagnostic biases. In addition, publication bias cannot be excluded, as studies reporting positive associations may be overrepresented in the literature. Finally, mechanistic interpretations remain limited, as most available data are indirect and observational. Together, these limitations underscore the exploratory character of this review and highlight the need for well-designed, prospective, and mechanistic studies to better elucidate the potential links between migraine and retinal pathology.

In summary, several retinal syndromes display features that may be common with migraine. However, the causal relationship between them still awaits verification, as the current evidence is often weak and internally inconsistent.

## Glossary

AMD: age-related macular degeneration;
ATG5: autophagy protein 5
CGRP: calcitonin gene-related peptide
CLR: calcitonin receptor-like receptor
CNS: central nervous system
CSD: cortical spreading depolarization
DDR: DNA damage response
GA: geographic atrophy
ipRGC: intrinsically photosensitive retinal ganglion cells
MTORC1: serine/threonine protein kinase mTOR 1
mtQC: mitochondrial quality control
NFE2L2: nuclear factor, erythroid 2 like 2
NTG: normal tension glaucoma
OCT: optical coherence tomography
OXPHOS: oxidative phosphorylation
PACAP: pituitary cyclase-activating polypeptide
PRKN: E3 ubiquitin-protein ligase parkin
PTA: posterior thalamic area
RAMP1: receptor activity modifying protein 1
RAO: retinal artery occlusion
RNFL: retinal nerve fiber layer
RONS: reactive oxygen and nitrogen species
RPE: retinal pigment epithelium
RVO: retinal vein occlusion
TRPA1: transient receptor potential cation channel subfamily A member 1
VEGFA: vascular endothelial growth factor A
